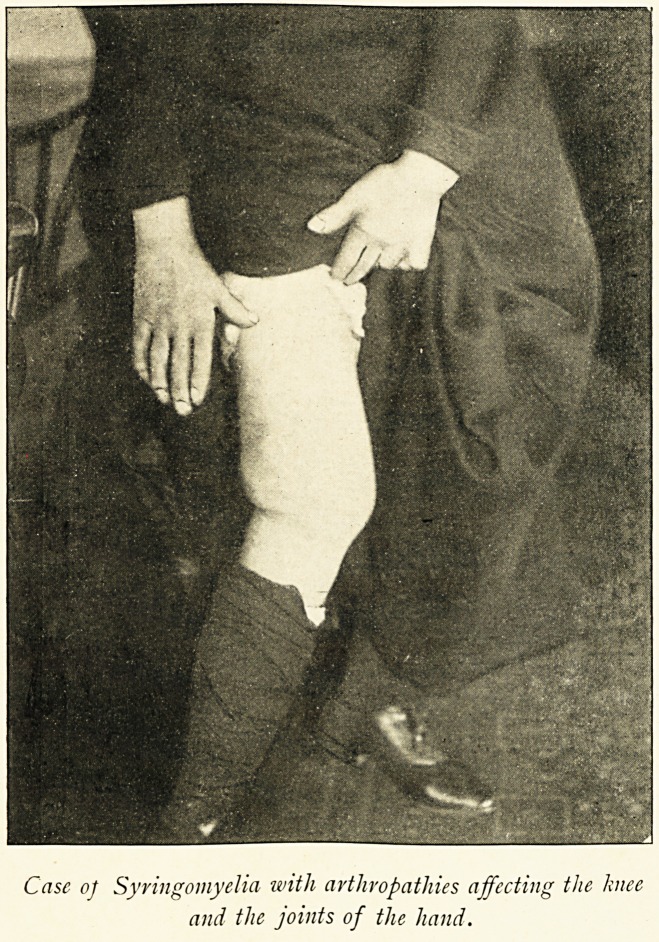# Cases of Joint Swellings Simulating Gout and Rheumatism

**Published:** 1905-06

**Authors:** R. Llewelyn Jones

**Affiliations:** Late Resident Medical Officer, Mineral Water Hospital, Bath


### CASES OF JOINT SWELLINGS SIMULATING GOUT AND RHEUMATISM.

BY

R. Llewelyn Jones, M.B. Lond.,

*Late Resident Medical Officer, Mineral Water Hospital, Bath.*

---

It has been wisely stated that the man who never makes mistakes never makes anything, and most of us will agree that the knowledge we gain from the appreciation of our errors is—though painful to our self esteem—much more likely to stick than if it had been acquired in a more pleasant manner. The following cases are examples of some of my own mistakes and those of other people.

Some two years ago I was consulted by a middle-aged man who had been invalided from Africa, supposed to be suffering from tuberculosis of the left apex. Bacilli had never been detected in his sputum, notwithstanding frequent examinations. His temperature for weeks was constantly raised at night. He had consulted in all some nine medical men, and during the attendance of the last he developed swelling of the right knee and ankle joints, which was deemed to be rheumatic in nature and for which he was sent to Bath. On examination I could detect no physical signs of phthisis. The joints referred to were swollen, and during my examination of them I happened to run my hand along the crest of his tibia, whereupon he complained of great tenderness, which I found to be due to a periosteal swelling, resembling a syphilitic node. I then ascertained that his wife had not accompanied him on his travels, that he had exposed himself to infection from a native woman, and had contracted a sore on his penis, which, however, was thought to be a soft chancre. On further examination I discovered some secondary syphilides. He had a well-marked corona veneris. I came to the conclusion that the joint swellings, the eruptions, and the pyrexia were specific in origin. I gave him iodides and mercurials, and his temperature rapidly dropped to normal; his joint and skin symptoms disappeared, and a year afterwards he was in good health. About the same time I had a similar case, but in this case the roseolous eruption and the joint swellings were ascribed to a gouty condition. In this case also a penile sore had been contracted a few months previously. Under antispecific treatment he mended rapidly.

These cases seem to me to justify Osler's comment, namely that we are not sufficiently alive to the fact that secondary syphilis may be attended by pyrexia of an intermittent or remittent type which may persist for months, and they also bear out the statement of Dr. Janeway, namely that such cases may be mistaken for tuberculosis.

It is quite easy to see that the two joint conditions may be readily confused, especially if syphilitic infection with secondary joint swellings should supervene in a patient already the subject of valvular heart disease. Such a case I heard of recently in which, fortunately, the development of a node led to early recognition.

The next case was that of a woman, aged 53, who was sent to Bath some four years ago for widespread rheumatic swelling of her joints. She was under my care for some time before I realised the nature of her case. My suspicion as to the correctness of the diagnosis was aroused by the fact that notwithstanding the marked joint disease she never complained of any pain. On enquiring into her personal history I found that eleven years previously her left knee swelled. It was practically painless, only it hurt her to go up and down stairs. It afterwards shrunk in size, and she suffered no inconvenience save on kneeling. The joints of her right hand, and then her left knee joint, went through the same sequence, painless swelling with deformity. Eight years ago she fell on her back, hurting her spine at the level of her shoulder blades, and a slight scar remained. On testing her sensory reactions I found that her tactile sensation was apparently normal, but her sense of pain and thermic sense were abolished over the greater part of her trunk and limbs. As regards her face, the skin of her forehead

*Case of Syringomyelia with arthropathies affecting the knee and the joints of the hand.*

seemed fairly normal, but the rest of her face, including the mucous membranes, was quite analgesic and thermo-anæsthetic. In front the skin of her thorax and abdomen showed the same alterations. Posteriorly the changes were all more marked on the right side. Over the skin covering the spinal column, at the lower dorsal and lumbar portion, she was, if anything, hyper-sensitive in all directions; otherwise, as regards the right half of her back, she was quite analgesic and thermo-anæsthetic. The scapular and lumbar reflexes were brisk, especially on right side. As regards her upper limbs, dissociated anæsthesia was almost universal, with the exception of an area over the flexor surface of the elbow joints. As regards her lower limbs, beyond the exception of a long strip of skin along the front of her thighs, the same conditions of dissociated anæsthesia prevailed. On the inner side of her legs were two raw patches presumably trophic, which, although objectively analgesic, were the seat of severe subjective pain. Her reflexes, deep and superficial, were apparently normal; there was occasionally incontinence of urine. As regards her muscles, there was little or no muscular wasting, save in the calves. I came to the conclusion that the condition was one of syringomyelia. She was afterwards seen by Risien Russell, who agreed with me as to the trophic origin of the joint changes, but he reminded me that dissociated anæsthesia might be met with in more pathological conditions than syringomyelia. Since then I understand that this peculiar phenomenon has been met with in pachymeningitis, tabes, and also in a case recently reported of neuritis of the brachial plexus. It might be thought that there was not much danger in confusing chronic rheumatism with syringomyelia, but a recent writer on spondylitis deformans, which is often diagnosed as rheumatism of the spine, states that it is not only possible but probable that such an error may occur. He lays stress on the fact that spinal curvature is common in syringomyelia, Friedrich's disease, and other nerve diseases. Furthermore, the symptom dissociated anæsthesia may be absent in syringomyelia, and on the other hand pain may be a prominent symptom. It seems to me, therefore, that this case emphasises the great importance of testing the sensory reactions in any case of joint disease, especially those of a chronic intractable form. Tabetic joints are also sometimes mistaken for rheumatic. Recently I saw such a case. Gowers has lately laid stress upon the importance of testing the knee jerks in cases of sciatica. Some twelve months ago I omitted this precaution, and shortly afterwards a spontaneous fracture gave a clue to the probable nature of the case.

Most observers will agree that it is wise to examine the rectum and pelvic organs in cases of sciatica or in any case of pain round the hip joints. At the Mineral Water Hospital I saw a case which was admitted with the diagnosis sciatica, and as far as this symptom was concerned it was perfectly correct. She had sciatica, but her pelvis was almost filled by a malignant growth. In the case of a lady I saw three years ago, the subject of gouty arthritis, the diagnosis of gout in the sacro-iliac joints was ventured upon, without a pelvic examination. Here the cause of the obstinate pain in her left sacral region turned out to be due to an acutely retroflexed uterus projecting into the rectum, and practically fixed by adhesions. Mr. Ransford, who saw the case with me, suggested the adoption of the genu-pectoral position when the pain was present. This she did and found the greatest benefit.

There is one cause of sciatic pain which might be laid more stress upon, namely arthritis of the lumbar spine. McCrae has recently laid stress on the importance of fixing the pelvis and ascertaining the power of motion in the lumbar vertebral joints. Another writer believes that osteoarthritis of the hip is often present. There is, I think, one point worthy of mention as regards spondylitis of the lumbar type. Recent research has taught us that an osteomyelitis of the vertebræ is by no means so rare as was thought. Such a process may depend upon a variety of organisms. In the so-called typhoid spine the bacillus typhosus has been discovered in the medulla of the vertebræ, and this in several cases, likewise pneumococci and other organisms. This seems to me to widen our view as to the ætiology of some cases of spondylitis deformans. Such cases may either end in suppuration or may not, and they may have as their main symptoms radiating pains in the lower limbs, with sensory alterations and muscular atrophy. It is quite easy to see, therefore, that such cases may be mistaken for lumbar neuritis, lumbago, sciatica, &c.

Now, while much stress has been laid upon referred pains in the lower extremities, too little, I venture to think, has been made of their varied significance in the head, neck, and upper extremities. The same precautions are equally necessary in the cervical as in the lumbar portion of the spinal column. I have no hesitation in stating that cervico-occipital and brachial neuralgias are often very early symptoms of cervical spondylitis. Such pains are, I am aware, usually ascribed to pressure on nerve roots, but in many instances they precede for months any limitation of movement, and are definitely paroxysmal. Again, such a condition may be readily confused with cervical meningitis of the hypertrophic type in its initial stages. I have had recently under my care a case illustrating the difficulties of differential diagnosis in this region. The patient, a young man, was said to be suffering from rheumatism of the spine. His cervical spine shows distinct swelling upon the left nuchal region, with much limitation of movement. He suffers with radiating pains down both arms, with some muscular atrophy. His arm jerks are apparently absent, his lower limb reflexes brisk, but no clonus or Babinski. His superficial reflexes are all absent, and he has widespread affection of sensation, especially as regards pain. His ocular reflexes are brisk, including the cilio-spinal; his left pupil is smaller than his right. His personal history revealed the fact that he had suffered with severe left-sided pleurisy. At present he shows marked retraction of the lung on that side, with displacement of the heart to the left axillary line, and the presence of systolic retraction just above his apex beat and also behind, near the angle of the scapula, suggest that he has an adherent pericardium as well. Such a case as this, though rare, certainly inculcates caution in diagnosing rheumatism of the spine. As to what the nature of the condition is it is certainly not easy to say, but I think that we may be sure of one thing, it is not rheumatism of the spine. The contraction of his left pupil led me to think of a central condition, but the fact that such affections of the sympathetic are not uncommon in apical pleurisies makes this symptom of uncertain value. Cervical caries with compression suggests itself, especially with the lung condition, but it is, I believe, unusual to have widespread affection of sensation without corresponding motor symptoms. He himself states that his legs are getting weaker.

Be the actual condition what it may, it serves to illustrate my contention that it is wiser to arrive at the diagnosis of gout and rheumatism by the slower and safer route of "Diagnosis by exclusion."

---

### A NEW SCHEME FOR INFANT MILK DEPÔTS.

BY

J. M. Fortescue-Brickdale, M.A., M.D.,

## Figures and Tables

**Figure f1:**